# Synthesis of Nano-Structured Conjugated Polymers with Multiple Micro-/Meso-Pores by the Post-Crosslinking of End-Functionalized Hyperbranched Conjugated Polymers

**DOI:** 10.3390/polym16091192

**Published:** 2024-04-24

**Authors:** Zhenfeng Liang, Hui Liang

**Affiliations:** Key Laboratory of Designed Synthesis and Application of Polymer Materials (DSAPM Lab), Key Laboratory for Polymer Composite and Functional Materials of Ministry of Education (PCFM Lab), School of Chemistry, Sun Yat-sen University, Guangzhou 510006, China; liangzhf7@mail2.sysu.edu.cn

**Keywords:** microporous conjugated polymer, hyperbranched conjugated polymer, post-crosslinking, Sonogashira coupling reaction, Heck reaction

## Abstract

A nano-structured conjugated polymer with multiple micro-/meso-pores was synthesized by post-crosslinking of an end-functionalized hyperbranched conjugated prepolymer. Firstly, an AB_2_ monomer 3-((3,5-dibromo-4-(octyloxy)phenyl)ethynyl)-6-ethynyl-9-octyl-9H-carbazole (PECz) was synthesized and polymerized by Sonogashira reaction to give the -Br end-functionalized hyperbranched conjugated prepolymer *hb*-PPECz. The photophysical and electrochemical properties of *hb*-PPECz were investigated. The λ_max_ of absorption and emission of *hb*-PPECz in tetrahydrofuran (THF) solution was 313 and 483 nm, respectively. The optical energy bandgap, highest occupied molecular orbital (HOMO), and lowest unoccupied molecular orbital (LUMO) energy levels of *hb*-PPECz were 2.98, −5.81, and −2.83 eV, respectively. Then, the prepolymer *hb*-PPECz was post-crosslinked by Heck reaction with divinylbenzene to give the porous conjugated polymer *c*-PPECz. The effects of *hb*-PPECz concentration and added dispersant polyvinylpyrrolidone (PVP K-30) on the morphology and porosity of *c*-PPECz were investigated. The resulting *c*-PPECzs showed multiple porous structures mainly constructed by micropores and mesopores. Under a higher *hb*-PPECz concentration (4 wt/v%), a bulky gel product was obtained. Under lower *hb*-PPECz concentrations (0.6 wt/v%~2 wt/v%), the resulting *c*-PPECzs were mainly composed of nano-sized particles. Nearly spheric nanoparticles (200~300 nm) (*c*-PPECz-5) were obtained under the concentration of 1 wt/v% in the presence of PVP (10 wt% of *hb*-PPECz). The Brunauer–Emmett–Teller (BET) surface area, pore volume, average pore size, and percentage of pore size below 10 nm of *c*-PPECz-5 were 10.7781 m^2^·g^−1^, 0.0108 cm^3^·g^−1^, 4.0081 nm, and 94.47%, respectively.

## 1. Introduction

Conjugated microporous polymers (CMPs) are a class of amorphous materials containing crosslinked conjugated backbones and a great amount of micropores. The combination of conjugated backbones and microporous structures provides not only excellent physicochemical properties but also great pore space to accommodate carriers. Moreover, the crosslinked structure can inhibit the dissolution of the materials. Therefore, CMPs exhibit many advantages over other organic microporous materials and have received increasing attention in the fields of clean energy [1,2,3], such as hydrogen storage materials [4,5,6], carbon dioxide capture and conversion [7,8,9,10], metal-ion rechargeable batteries [11,12], supercapacitors [13,14], heterogeneous catalysts [15,16], light-harvesting materials [17,18], luminescent materials [19,20], and photovoltaic materials [21,22].

Almost all the reported CMPs were synthesized by A_m_ + B_n_ small-molecule monomer systems. The structure and functionality of A_m_ and B_n_ were designed to control the structure and photoelectric properties of CMPs, including the morphology, construction of a porous structure, porosity, and energy gap [2]. CMPs synthesized by A_m_ + B_n_ small-molecule monomer systems are typically irregular powder materials and restrict further applications. Other forms of CMPs with nanospherical, nanotubular, thin-film structures were reported to be used in high-performance energy-related applications [2]. Nanospherical CMPs have promising applications in gas storage separation, energy storage, and solar energy conversion. However, the synthesis of nanospherical CMPs is still a great challenge owing to the poor dispersibility and solution processing of CMPs.

**Scheme 1:**
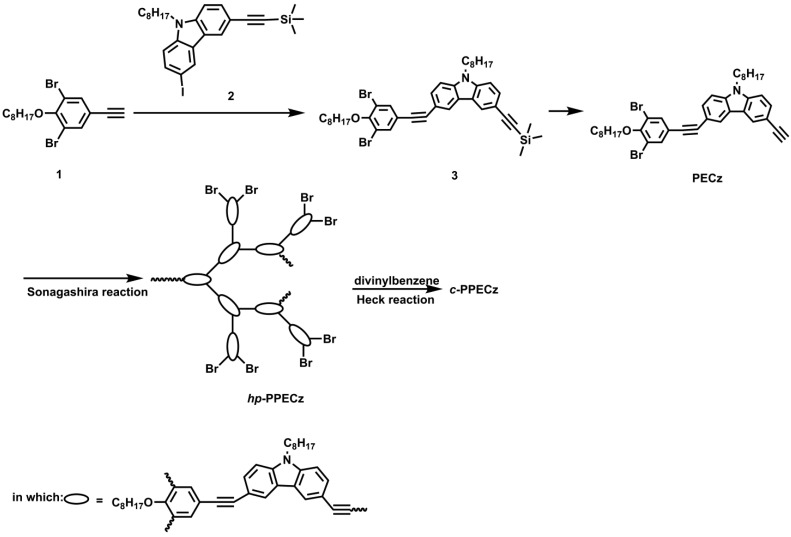
Structure and synthetic route of hyperbranched prepolymer (*hb*-PPECz) and porous crosslinked conjugated polymer (*c*-PPECz).

Herein, we reported the synthesis of a nano-structured micro-/meso-porous crosslinked conjugated polymer by the post-crosslinking of a -Br end-functionalized hyperbranched conjugated prepolymer via Heck reaction with divinylbenzene. The structures and synthetic route of hyperbranched prepolymer and porous crosslinked conjugated polymer are shown in Scheme 1.

Firstly, an AB_2_-type monomer (3-((3,5-dibromo-4-(octyloxy)phenyl)ethynyl)-6-ethynyl-9-octyl-9H-carbazole, PECz) constructed by phenyl-, ethynyl-, and carbazole-blocks was synthesized and polymerized to give the hyperbranched conjugated prepolymer *hb*-PPECz. *hb*-PPEcz carries -Br group in each branch end. Post-crosslinking of *hb*-PPECz by Heck reaction with divinylbenzene gave crosslinked conjugated polymer *c*-PPECz. It can be expected that porous structures resulting from the free volume between the conjugated branches and crosslinked conjugated skeleton will form in *c*-PPECz. Moreover, using a hyperbranched prepolymer instead of the small molecule as the crosslinking block will result in better solubility of the former, which will generate a swellable gel layer on the surface of the precipitated product. This surface gel layer can act as a volume-repulsion stabilizer to prevent the agglomeration of gel particles and make it possible to obtain better dispersibility of the resulting *c*-PPECz, resulting in spherical products like in the case of radical precipitation/dispersion polymerization [23,24]. In radical dispersion polymerization, the addition of a dispersant can inhibit agglomeration between the particles better than in radical precipitation polymerization without a dispersant. Therefore, the effect of the added dispersant of PVP K-30 on the morphology and pore structure of the post-crosslinking particles was investigated. By varying the conditions of the post-crosslinking reaction, nano-structured nearly spherical multiple micro-/meso-porous *c*-PPECz with a moderate surface area were obtained. On the other hand, the abundant coordination sites (including C≡C bonds and O, N heteroatoms) provide *c*-PPECz with the ability to complex with metal ions to increase the conductivity and content of active metal species, which can improve the performance of CMPs as electrode materials and electrocatalysts. Therefore, the loading of metal ions into *c*-PPECz was investigated.

## 2. Experimental Section

### 2.1. Materials and Instruments

N-iodosuccinimide (98%), 1-bromooctane (98%), 1,4-diiodobenzene (98%), trimethylsilylacetylene (98%), 2,6-dibromophenol (98%), copper(I) iodide (98%), and bis(triphenylphosphine)palladium chloride (98%) were purchased from Acros Organics. Sodium sulfate anhydrous (99%), sodium chloride (99.5%), potassium hydroxide (95%), 3,6-diiodocarbazole (98%), 2,7-diiodofluorene, palladium acetate (99%), (O-tolyl)_3_P (97%), and diethylbenzene (80% with stabilizer) were purchased from Shanghai Macklin Biochemical (Shanghai, China); dimethyl sulfoxide (99%), ethyl acetate (99%), tetrahydrofuran (99%), methanol (99%), triethylamine (99%), and petroleum ether (99%) were purchased from Shanghai Aladdin Biochemical Technology Co., Ltd. (Shanghai, China) PVP K-30 was purchased from Guangdong Zhuguang New Energy Technology Co., Ltd. (Guangzhou, China) Other reagents were all reagent-grade materials and purified by standard methods if needed.

The scanning electron microscope (SEM) used is the Hitachi High-Technologies S-4800 model (Tokyo, Japan), with an acceleration voltage of 5 kV. The average molecular weight (Mn) and molecular weight distribution (Mw/Mn) of the polymer were determined using an Agilent Technologies PL-GPC 50 gel permeation chromatography system (Bournemouth, UK). The GPC column configuration consisted of an Agilent PL 10 μM MIXED-B gel column followed by two Agilent PL 5 μ MMIXED-D gel columns in series. Polystyrene (PS) was used as a standard sample, and detection was performed with a refractive index detector. The test temperature was maintained at 40 °C, tetrahydrofuran (THF) was used as the mobile phase, and the flow rate was set at 1.0 mL/min.

^1^H NMR spectra were recorded on an Avance III 400 MHz NMR spectrometer (Bruker, Fällanden, Switzerland) in CDCl_3_ with tetramethylsilane as an internal standard. Chemical shifts were reported in ppm relative to the internal standard. The cyclic voltammograms were recorded on an IM6e electrochemical workstation (German ZAHNER Co., Ltd., Kronach, Germany). The three-electrode system used involved a glassy carbon electrode as the working electrode, calomel electrode as the reference electrode (RE), platinum electrode as the auxiliary electrode (AE), scanning speed of 20 mv/s, polymer sample concentration of 10^−4^ mol/L, using dichloromethane as the solvent, and adding tetrabutylhexafluorophosphate ammonium (0.1 M dichloromethane solution) as the supporting electrolyte.

The instrument used for adsorption–desorption analysis is the Belsorp instrument model BSD-660M A6B3M (Belsorp Instrument Technology Co., Ltd, Beijing, China) series fully automatic surface area and pore size analyzer. The instrument is capable of measuring pore sizes ranging from 3.5 to 5000 angstroms. UV–vis absorption spectra were recorded on a CARY5000 spectrophotometer. Fluorescence spectra were recorded on a Steady State and Transient Fluorescence Spectrometer-1000 (Edinburgh Company, Edinburgh, UK) at an excitation wavelength of 313 nm.

### 2.2. General Procedures of Syntheses

Compound **1** [25] and Compound **2** [26] were synthesized by the procedures in the literature, respectively.

Synthesis of Compound **3**: 2 g (5.16 mmol) of Compound **1**, along with 91 mg of Pd(PPh_3_)_2_Cl_2_ and 39 mg of CuI, were added to a 100 mL round-bottom flask, evacuated, and filled with nitrogen gas, and this process was repeated three times. Under nitrogen protection, 5 mL of freshly distilled THF and 1.1 mL (7.96 mmol) of Et_3_N were added, followed by slow dropwise addition of 2.63 g (5.16 mmol) of Compound **2** dissolved in 5 mL of THF, with a controlled drip time within one hour. After stirring the reaction at room temperature for 24 h, the reaction mixture was filtered through a short column of silica gel to remove the precipitate. The filtrate was rotary evaporated to remove the solvent and purified by petroleum ether through a silica gel column. This yielded 3.85 g of product (colorless transparent liquid) with a yield of 98%. ^1^H NMR δ (ppm): 8.48 (s, 2H), 7.69 (s, 2H), 7.54 (d, 1H), 7.52 (d, 1H), 7.40 (dd, 1H), 7.34 (dd, 1H), 4.27 (t, 2H), 4.01 (t, J = 6 Hz, 2H), 1.87 (*p*, J = 6 Hz, 4H), 1.65–1.25 (m, 20H), 0.91 (t, J = 6 Hz, 6H), and 0.24 (s, 9H).

Synthesis of PECz: 8.30 g (10.9 mmol) of Compound **3** was added to a 250 mL two-neck flask, evacuated, and filled with nitrogen gas, repeating this process three times. Separately, a mixture of 8 mL tetrahydrofuran and 44 mL methanol solvent was bubbled with nitrogen gas to remove oxygen for 30 min and then added to the aforementioned two-neck flask. After thorough mixing and dissolution, 3.3 mL of 20% KOH aqueous solution was added. The reaction proceeded at room temperature under nitrogen protection for 2 h. Upon completion of the reaction, the mixture was filtered through a short column of silica gel, followed by rotary evaporation to remove the solvent. The crude product was purified using petroleum ether through a silica gel column. This yielded 7.36 g of white crystals with a yield of 98%. ^1^H NMR δ (ppm): 8.48 (s, 2H), 7.69 (s, 2H), 7.54 (d, 1H), 7.52 (d, 1H), 7.40 (dd, 1H), 7.34 (dd, 1H), 4.27 (t, 2H), 4.05 (t, J = 6 Hz, 2H), 3.28 (s, 1H), 1.87 (*p*, J = 6 Hz, 4H), 1.65–1.25 (m, 20H), and 0.91 (t, J = 6 Hz, 6H).

Synthesis of *hb*-PPECz: 19.8 mg of 1,2-diiodobenzene (0.06 mmol), 63 mg of Pd(PPh_3_)_2_Cl_2_, 48 mg of triphenylphosphine, and 42 mg of CuI were added to a 25 mL two-neck flask equipped with a dropping funnel, and the process of vacuum evacuation and nitrogen gas purging was repeated three times. A mixture of 7.5 mL of toluene and 7.5 mL of triethylamine, degassed by three freeze–pump–thaw cycles, was added. The resulting reaction mixture was heated to 90 °C. Then, a solution of 2.07 g (3 mmol) PECz in 7.5 mL toluene, degassed by three freeze–pump–thaw cycles, was added dropwise to the reaction mixture at a rate of 1 drop per 40 s. After stirring for 48 h, the reaction mixture was filtered through a short silica gel column. The filtrate was then rotary evaporated to remove the solvent. Subsequently, 10 mL of chloroform was added to dissolve the solid, and then methanol was used to precipitate and separate the polymer *hb*-PPECz. The polymer was purified by two rounds of dissolution and precipitation, yielding a tan solid weighing 1.01 g.

Synthesis of *c*-PPECz-1: 100 mg of *hb*-PPECz, 8 mg of palladium acetate, and 13.4 mg of (O-tolyl)_3_P were added to a 25 mL single-neck flask, and the process of vacuum evacuation and nitrogen purging was repeated three times. Then, a solution of 5 mL toluene, 0.4 mL triethylamine, and 0.0163 g divinylbenzene, degassed by three freeze–pump–thaw cycles, was added. The reaction mixture was then heated to 90 °C and allowed to react for 48 h. After cooling to room temperature under stirring, the reaction mixture was added dropwise to methanol to precipitate the product. The product was dried under vacuum to give *c*-PPECz-1, 0.095 g.

Synthesis of *c*-PPECz-2~*c*-PPECz-5: *c*-PPECz-2~*c*-PPECz-4 were synthesized under the same conditions of *c*-PPECz-1 except for the concentration of *hb*-PPECz. *c*-PPECz-5 was synthesized under the same conditions as *c*-PPECz-3 except for the addition of 10 mg PVP K-30.

## 3. Results and Discussion

### 3.1. Synthesis of Hyperbranched Conjugated Prepolymer hb-PPECz

**Figure 1:**
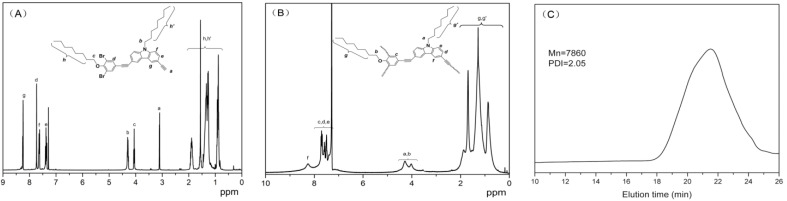
(**A**) ^1^H NMR spectrum of AB_2_ monomer PECz; (**B**) ^1^H NMR spectrum of hyperbranched prepolymer *hb*-PPECz; (**C**) GPC curve of hyperbranched prepolymer *hb*-PPECz.

As shown in Scheme 1, an AB_2_-type monomer (3-((3,5-dibromo-4-(octyloxy)phenyl)ethynyl)-6-ethynyl-9-octyl-9H-carbazole, PECz) containing one ethynyl group and two -Br groups was synthesized. Due to the significant reactivity difference between -Br and -I groups, the Sonogashira reaction of Compound **1** with Compound **2** selectively occurred between ethynyl and -I groups, leaving the -Br groups unreacted. With sequential deprotection, the AB_2_-type monomer PECz was obtained. The ^1^H NMR spectrum of PECz and peak assignments are shown in Figure 1A. Peaks corresponding to the protons of -C≡CH(a), -N-CH_2_-(b), -OCH_2_-(c), phenyl(d, e, f, g), and –(CH_2_)_6_CH_3_(h, h’) can be observed. The integral ratio of a/b/c/d/e/f/g = 1/2/2/2/2/2/2, which is consistent with the theoretical value, indicating the successful synthesis of monomer PECz.

The monomer PECz was polymerized by the Sonogashira reaction to give the hyperbranched conjugated prepolymer *hb*-PPECz. For the polymerization of AB_n_ monomer to give hyperbranched polymer, core molecules were usually added to control the product’s molecular weight by varying the molar ratio of [AB_n_ monomer]/[core molecule] and obtain a narrower molecular weight distribution [25]. Therefore, 1,4-diiodolbenzene was added as the core molecule. The ^1^H NMR spectrum of *hb*-PPECz and its peak assignments are shown in Figure 1B. No signal of -C≡CH can be observed, indicating the complete conversion of -C≡CH. As reported in a previous paper on the synthesis of hyperbranched poly (*m*-phenyleneethynylene-*alt*-*p*-phenyleneethynylene) (*hb*-PMPE) [25], the signal of -OCH_2_- in dendritic (D), terminal (T), and linear (L) units of *hb*-PMPE can be distinguished as three separated peaks within the range of δ 4.0~4.4 ppm in the ^1^H NMR spectrum, since the chemical environment of the alkoxyl group in D, T, and L units of *hb*-PMPE is different from each other: in the D unit, both the two o-substituent groups of alkoxyl are ethynylene groups; in the T unit, both the two o-substituent groups of alkoxyl are bromide groups; while in the L unit, one o-substituent group is ethynylene and another is bromide. Therefore, the branching degree (DB) of *hb*-PMPE can be calculated from the ^1^H NMR spectrum by the integration of these three peaks. But for *hb*-PPECz, we cannot calculate the DB from the ^1^H NMR spectrum since the signals of –OCH_2_- in D, T, and L units are partially overlapped by the signal of -NCH_2_-. The GPC curve of *hb*-PPECz is shown in Figure 1C, and the curve exhibits a unimodal distribution. *hb*-PPECz with Mn of 7860 and PDI of 2.05 was obtained.

### 3.2. Photophysical and Electrochemical Properties of Hyperbranched Prepolymer hb-PPECz

**Figure 2:**
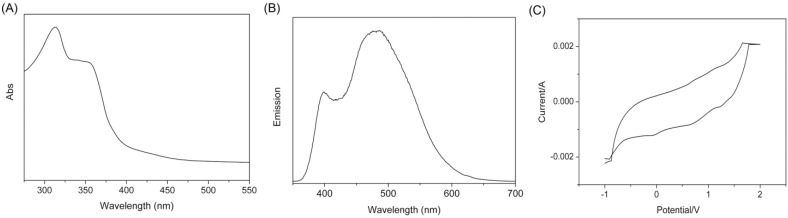
(**A**) Normalized UV-vis absorption spectrum of *hb* −PPECz in THF solution. (**B**) Normalized luminescent spectrum of *hb*-PPECz in THF solution (λ_ex_ = 313 nm). (**C**) Cyclic voltammetry curve of *hb*-PPECz in CH_2_Cl_2_ solution.

The photophysical and electrochemical properties significantly affect the performance of CMPs for applications in the fields of clean energy [27]. To evaluate the potential application in the field of clean energy, the photophysical and electrochemical properties of *hb*-PPECz were investigated by UV-vis absorbance, luminescence, and cyclic voltammetry (CV), respectively. The normalized UV-vis absorbance and luminescence (λ_ex_ = 313 nm) spectra of *hb*-PPECz are shown in Figure 2A,B, respectively. The UV-vis absorption spectrum shows a major band (λ_max_ at 313 nm) with a significant shoulder peak (λ_sh_ at 345 nm). The luminescence spectrum of *hb*-PPECz shows a major band with λ_max_ at 483 nm, emitting blue light under 365 nm irradiation. The optical energy bandgap (E_g_^opt^) can be estimated from the onset absorption wavelength (λ_onset_) by the formula E_g_^opt^ = 1240/λ_onset_. The E_g_^opt^ of *hb*-PPECz was 2.98 eV.

To gain further insight into the electronic properties, cyclic voltammetry (CV) measurements were conducted to reveal the energy band structure of *hb*-PPECz. The CV curve of *hb*-PPECz is shown in Figure 2C. HOMO and LUMO energy levels of *hb*-PPECz were −5.81 eV and −2.83 eV, which werecalculated from its oxidation potential ($E_{pa(onset)}$ = 1.05 eV) by the following formulas:

(1) $$E_{HOMO}=-4.84\;\mathrm{eV}-E_{pa(onset)},$$

(2) $$E_{LUMO}=E_{g}^{opt}-\left|E_{HOMO}\right|$$

in which $E_{HOMO}$ stands for the HOMO energy level, $E_{LUMO}$ stands for the LUMO energy level, and $E_{pa(onset)}$ stands for the onset oxidation potential. The standard hydrogen electrode (NHE) potential is referenced to the vacuum energy level as −4.5 eV, and in this experiment, a mercury–silver amalgam electrode (in a 0.1 M KCl solution) is used as the reference electrode, with a potential relative to NHE of 0.34 eV.

A low energy gap is a desired characteristic for high-performance CMPs. A lower energy gap means longer conjugation length and higher conductivity. Due to the long conjugation length composed of phenyl, ethynyl, and carbazole units for *hb*-PPECz, the energy gap of *hb*-PPECz is relatively low and makes *hb*-PPECz potential applications in clean energy areas such as electrode materials and electrocatalysts.

### 3.3. Synthesis and Pore Structure Characterization of Multiple Micro-/Meso-Porous Conjugated Polymer c-PPECz

The hyperbranched prepolymer *hb*-PPECz carries an aromatic -Br group in each of its branch ends. Therefore, post-crosslinking of *hb*-PPECz was carried out by Heck reaction with divinyl benzene (DVB) to give a crosslinked conjugated polymer (*c*-PPECz). Due to the free volume between the branches of *hb*-PPECz and crosslinked conjugated skeleton, *c*-PPECz should contain micro-/meso-pores. The effects of the concentration of *hb*-PPECz and added dispersant (PVP K-30) on the morphology and porosity of *c*-PPECz were investigated. The post-crosslinking conditions and product appearance are shown in Table 1. With a higher concentration of *hb*-PPECz (4 wt/v%), the bulky gel was obtained. Within the range of 0.6 wt/v%~2 wt/v%, turbid dispersions were obtained.

**Figure 3:**
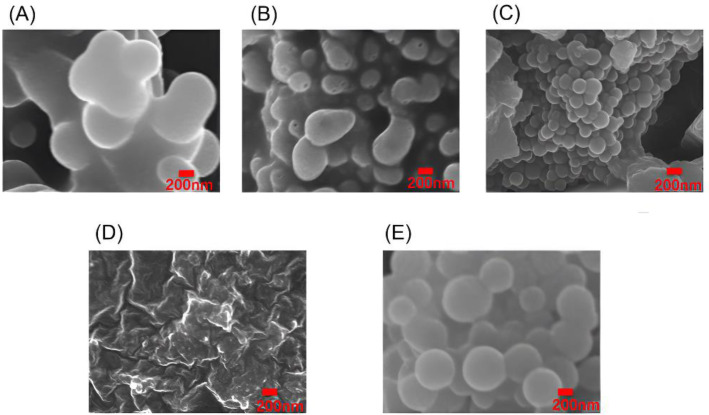
SEM images of crosslinked conjugated polymer *c*-PPECzs: (**A**) *c*-PPECz-1; (**B**) *c*-PPECz-2; (**C**) *c*-PPECz-3; (**D**) *c*-PPECz-4; (**E**) *c*-PPECz-5.

The SEM images of *c*-PPECzs are shown in Figure 3. Except for *c*-PPECz-4 (Figure 3D), the other four *c*-PPECzs were mainly composed of nano-sized particles, with a particle size of approximately 200~300 nm; however, significant bonding between the particles can be observed in Figure 3A–C. By comparing Figure 3A–C, there is no significant change in the particle size with increasing concentration of *hb*-PPECz. However, a significant effect of concentration on the shape regularity of the particles can be observed. Under the concentration of 1 wt/v%, spherical-like particles (*c*-PPECz-3) were obtained. In radical dispersion polymerizations [24,28], a dispersant was added to prevent aggregation and control the morphology of polymerization products. Therefore, the effect of dispersant PVP K-30 on the morphology of *c*-PPECz was investigated under the concentration of 1 wt/v%. The result showed that by the addition of PVP (10 wt% of *hb*-PPECz), the shape of product particles became more regular, and most of the particles were nearly spherical (Figure 3E).

**Figure 4:**
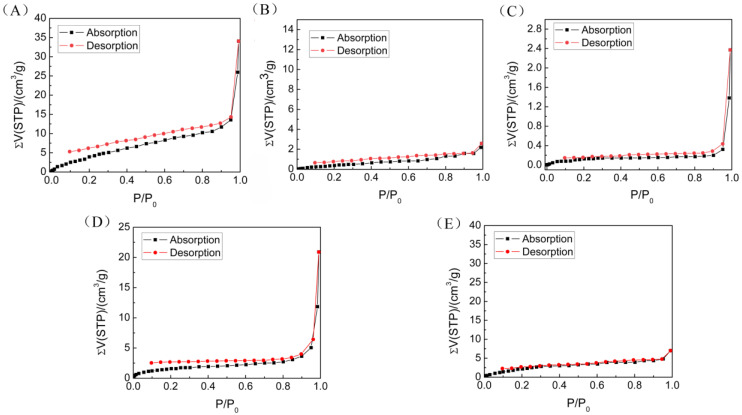
N_2_ adsorption and desorption curves of *c*-PPECzs: (**A**) *c*-PPECz-1; (**B**) *c*-PPECz-2; (**C**) *c*-PPECZ-3; (**D**) *c*-PPECz-4; (**E**) *c*-PPECz-5.

**Figure 5:**
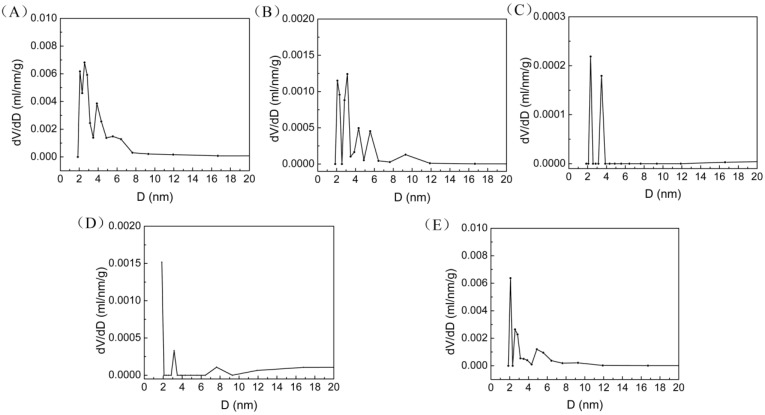
Pore size distribution curves of *c*-PPECzs: (**A**) *c*-PPECZ-1; (**B**) *c*-PPECz-2; (**C**) *c*-PPECz-3; (**D**) *c*-PPECz-4; (**E**) *c*-PPECz-5.

The pore structure and microporous characteristics of *c*-PPECzs were investigated by the N_2_ adsorption–desorption isotherms at a temperature of 77.4 K, as shown in Figure 4. The vertical axis of the adsorption–desorption isotherm is represented by ∑V(STP) (cm^3^/g), indicating the adsorption amount under standard conditions. It can be observed that the adsorption curve of each *c*-PPECz shows a large slope at low pressure (P/P_0_ < 0.1), exhibiting typical characteristics of microporous materials. Based on the adsorption–desorption isotherm data, pore structure information of the sample is obtained through computational analysis, and the pore size distribution is determined using the BJH (Barret–Joyner–Halenda) method. The BET equation is employed to determine the specific surface area of the sample, while the total pore volume and average pore diameter are calculated from the nitrogen adsorption amount at a relative pressure of 0.990. The BET specific surface area, total pore volume, average pore diameter, and the percentage of pore size below 10 nm for the *c*-PPECzs are presented in Table 2. Additionally, based on the N_2_ adsorption–desorption isotherms, pore size distribution curves are obtained using the Barret–Joyner–Halenda method, as illustrated in Figure 5. The vertical axis, represented by dV/dD (mL/nm/g), signifies the increment of the pore volume.

Comparing *c*-PPECz-1 with *c*-PPECz-4 shows that under a higher *hb*-PPECz concentration of 4 wt/v%, the reaction exhibits a gel phenomenon, the pore area percentage is smaller, and the specific surface area is reduced. Comparing *c*-PPECZ-3 with *c*-PPECz-5 shows that the addition of dispersant PVP K30 results in smaller pore size, higher pore content, and a significant increase in specific surface area. The results showed that the addition of PVP K-30 was not only conducive to obtaining spherical particles but also conducive to increasing the micropore/mesopore content and specific surface area. Moreover, the results show that *c*-PPECzs have multiple porous structures mainly constructed by micropores and mesopores. This multiple porous structure is particularly beneficial for electrode materials. Micropores promote charge enrichment, while mesopores promote charge transfer, electrolyte penetration, and mass transfer [29].

### 3.4. Loading of Metal Ion in c-PPECz

**Figure 6:**
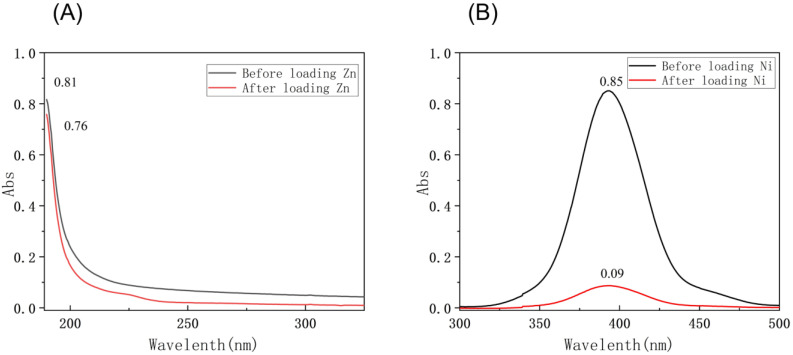
UV spectra of metal ion solutions: (**A**) Zn(II); (**B**) Ni(II).

For certain applications, doping of metal ions in CMPs can significantly improve the performance of CMPs [30,31,32]. The obtained *c*-PPECz contains abundant coordination sites, including C≡C bonds and heteroatoms of O and N. These coordination sites can easily bind with metallic ions and make them homogeneously dispersed in CMPs, hence improving the performance of CMPs as electrode materials and electrocatalysts by increasing the conductivity and content of active metal species of CMPs [33]. Therefore, as examples, the loading of Zn^2+^ and Ni^2+^ ions in *c*-PPECz was investigated. ZnCl_2_ and NiSO_4_ were dissolved in distilled water to prepare a metal ion solution with a concentration of 400 mg·L^−1^. A total of 10 mg of *c*-PPECz-5 was suspended in 20 mL of the metal ion solution. The mixture was then sealed and allowed to stand at room temperature for 24 h. After adsorption, the adsorbent was separated from the solution by filtration, and the filtrate was collected for analysis using a UV/visible spectrophotometer. The results are shown in Figure 6. The adsorption capacity $q_{e}$ (mg·g^−1^) was calculated according to the following formula:

(3) $$q_{e}=\frac{V\times (c_{0}-c_{e})}{W}$$

in which V represents the volume of the metal ion solution (L), $c_{0}$ represents the initial concentration of the metal ion solution (mg·L^−1^), $c_{e}$ represents the concentration of the metal ions after adsorption (mg·L^−1^), and W represents the mass of *c*-PPECz (g). The adsorption capacity of *c*-PPECz-5 for Zn(II) was 206 mg·g^−1^, and for Ni(II), it was 523 mg·g^−1^.

## 4. Conclusions

A nano-structured conjugated polymer (*c*-PPECz) with multiple micro-/meso-pores was synthesized by the post-crosslinking Heck reaction of DVB with *hb*-PPECz, which was synthesized by an AB_2_ monomer (3-((3,5-dibromo-4-(octyloxy)phenyl)ethynyl)-6-ethynyl-9-octyl-9H-carbazole, PECz). The *hb*-PPECz concentration and added dispersant PVP K30 in the post-crosslinking reaction showed significant effects on the morphology and porosity of *c*-PPECz. Lower *hb*-PPECz concentration was beneficial for obtaining nano-structured products. The addition of dispersant PVP K-30 was not only conducive to obtaining spherical particles but also conducive to increasing the micropore/mesopore content and specific surface area. Nearly spheric nanoparticles (200~300 nm) (*c*-PPECz-5) with multiple porous structures mainly constructed by micropores and mesopores were obtained under the concentration of 1 wt/v% in the presence of PVP K-30 (10 wt% of *hb*-PPECz). The combination of multiple micro-/meso-porous structures, relatively low energy gap, and abundant coordination sites provides the obtained *c*-PPECzs with potential applications in the fields of clean energy, such as electrode materials and electrocatalysts.

## Figures and Tables

**Table 1:** Post-crosslinking conditions for Heck reaction of *hb*-PPECz with DVB.

	*c*-PPECz-1	*c*-PPECz-2	*c*-PPECz-3	*c*-PPECz-4	*c*-PPECz-5 *
[*hb*-PPECz] (wt/v%)	2	0.6	1	4	1
product appearance	dispersion	dispersion	dispersion	bulky gel	dispersion

Other conditions: *hb*-PPECz, 0.1 g; DVB, 0.0134 g; palladium acetate, 8 mg; (O-tolyl)_3_P, 13.4 mg; in Tol; 90 °C; 48 h. * Added PVP K-30: 10 wt% of *hb*-PPECz.

**Table 2:** Pore characteristics of *c*-PPECzs.

	BET Specific Surface Area/(m^2^·g^−1^)	Pore Volume/(cm^3^·g^−1^)	Average Pore Size/nm	Percentage of Pore Size below 10 nm
*c*-PPECz-1	15.7014	0.0476	12.1263	83.24%
*c*-PPECz-2	1.4085	0.0034	9.6557	93.72%
*c*-PPECz-3	0.3207	0.0032	39.9100	54.39%
*c*-PPECz-4	5.9429	0.0306	20.5960	58.73%
*c*-PPECz-5	10.7781	0.0108	4.0081	94.47%

## Data Availability

Data are contained within the article.
